# Supplementary material for: Lipid profiling reveals unsaturated lipid reduction in women with Alzheimer's disease
Source: Alzheimers Dement. 2025 Aug 20;21(8):e70512. doi: 10.1002/alz.70512 (PMC12365783; doi:10.1002/alz.70512)
Supplement: Supplementary file 2 — Supporting Information [file ALZ-21-e70512-s005.pdf]

Supplementary figure 1

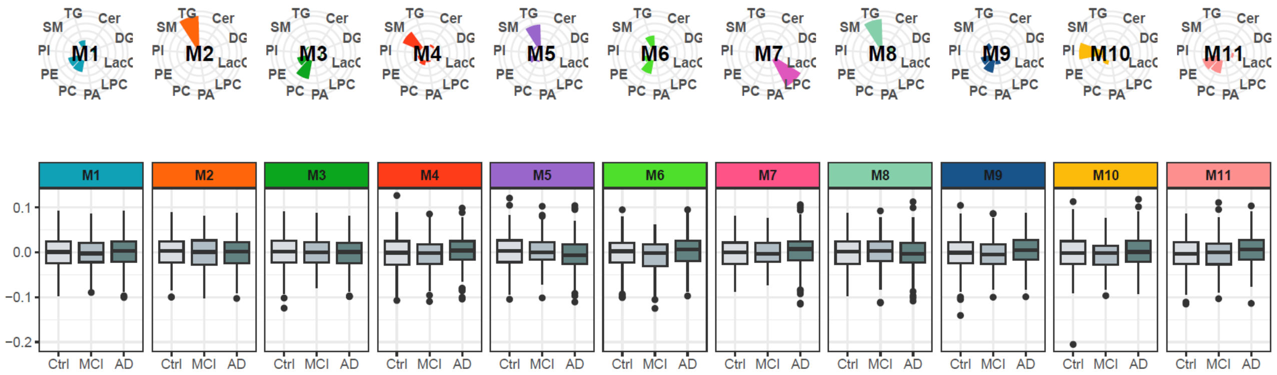

Supplementary figure 1. Full module composition and Eigengene boxplots by AD status and Sex. Percentwise lipid composition of each module, along with eigengene boxplot by cognition status (Ctrl = cognitively healthy, MCI = mild cognitive impairment, and AD = Alzheimer's disease).
