# Supplementary material for: Lipid profiling reveals unsaturated lipid reduction in women with Alzheimer's disease
Source: Alzheimers Dement. 2025 Aug 20;21(8):e70512. doi: 10.1002/alz.70512 (PMC12365783; doi:10.1002/alz.70512)
Supplement: Supplementary file 3 — Supporting Information [file ALZ-21-e70512-s007.pdf]

Supplementary figure 2

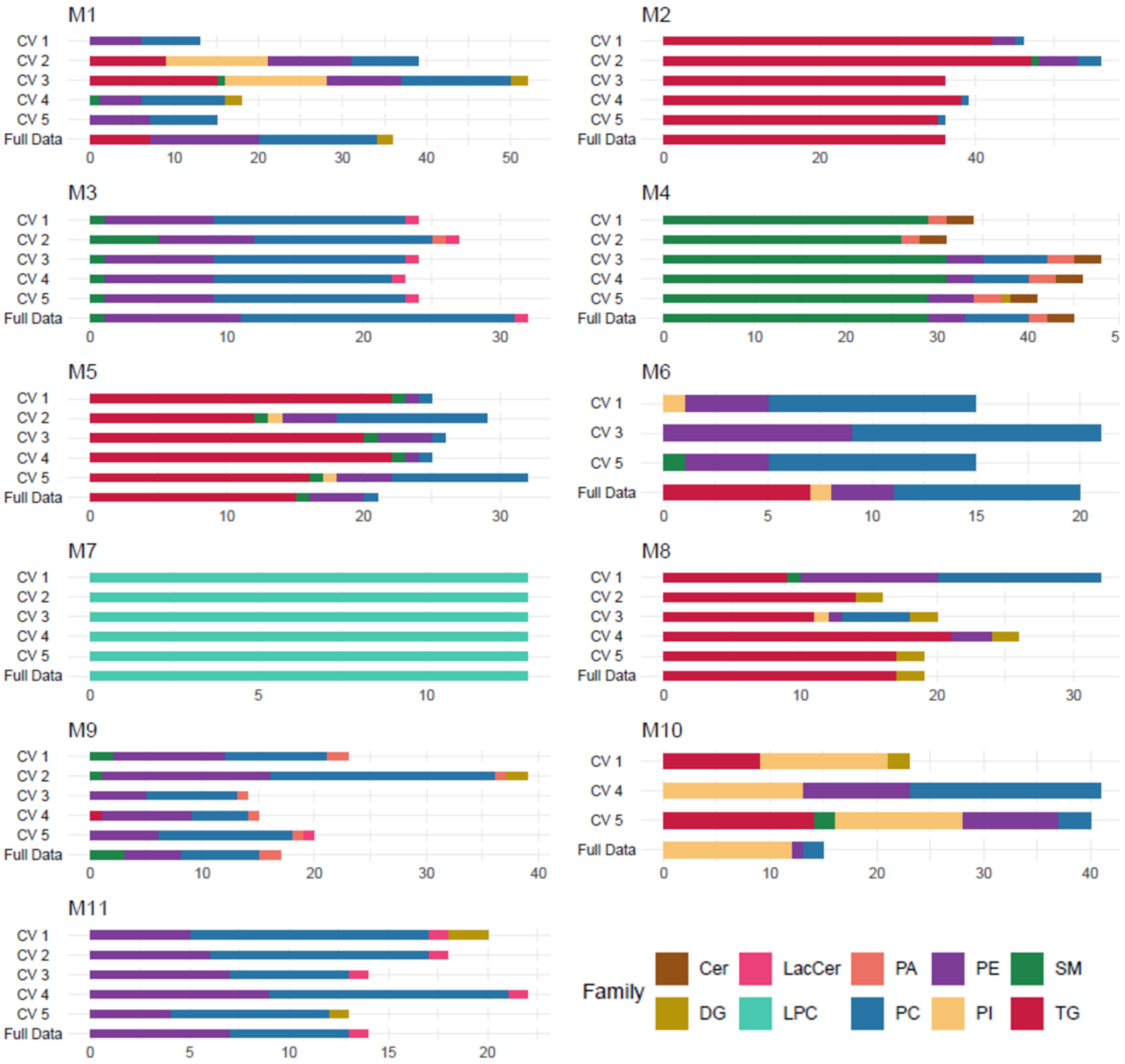

Supplementary figure 2. Lipid composition of WCNA modules in 5-fold cross validation compared to the full data set, each cross validation set consisting of 4/5th of the data.
