# Supplementary figures and images for: Lipid profiling reveals unsaturated lipid reduction in women with Alzheimer's disease
Source: Alzheimers Dement. 2025 Aug 20;21(8):e70512. doi: 10.1002/alz.70512 (PMC12365783; doi:10.1002/alz.70512)

Supplementary figure 3

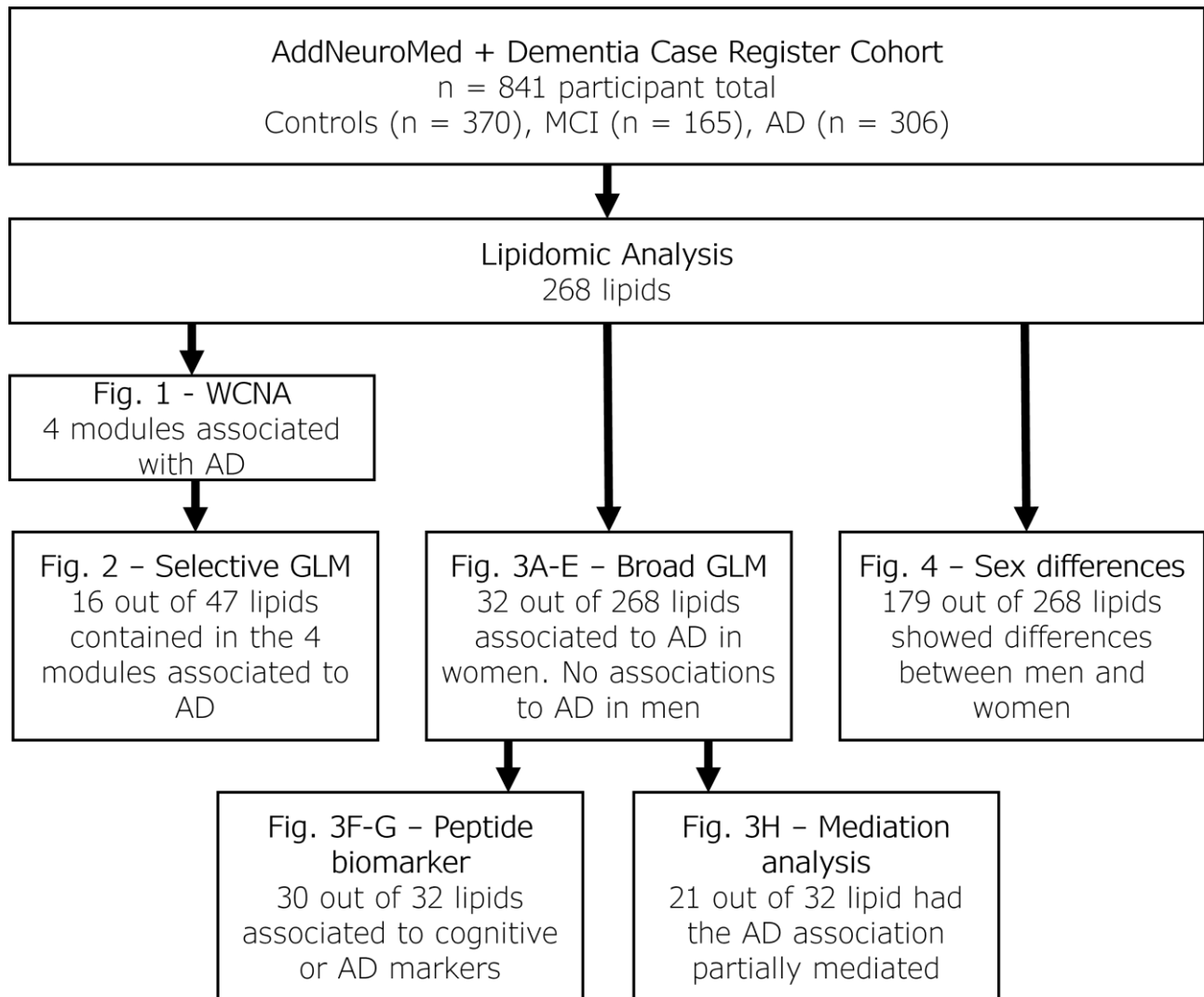

Supplementary figure 3. Study overview

Supplement: Supplementary file 4 — Supporting Information [file ALZ-21-e70512-s001.pdf]
